# Heterologous expression and characterization of a 3-ketosteroid-∆^1^-dehydrogenase from *Gordonia neofelifaecis* and its utilization in the bioconversion of androst-4,9(11)-dien-3,17-dione

**DOI:** 10.1007/s13205-017-0601-4

**Published:** 2017-04-08

**Authors:** Weiyi Wang, Fanglan Ge, Caihong Ma, Jiang Li, Yao Ren, Wei Li, Jinsong Fu

**Affiliations:** College of Life Sciences, Sichuan Normal University, Chengdu, 610061 People’s Republic of China

**Keywords:** *Gordonia neofelifaecis* NRRL B-59395, 3-Ketosteroid- ∆1-dehydrogenase, Bioconversion, Androst-1,4,9(11)-trien-3,17-dione

## Abstract

3-Ketosteroid-∆^1^-dehydrogenase (KstD), a key enzyme in microbial steroid catabolism, catalyzes the *trans*-axial elimination of the C1 and C2 hydrogen atoms of the A-ring from the polycyclic ring structure of 3-ketosteroids, and it was usually used to transform androst-4-ene-3,17-dione (AD) to produce androsta-1,4-diene-3,17-dione. Here, the KstD from *Gordonia neofelifaecis* was expressed efficiently in *Escherichia coli*. *E. coli* cells expressing KstD_3gor_ were subjected to the investigation of dehydrogenation activity for different steroids. The results showed that KstD_3gor_ has a clear preference for steroid substrates with 3-keto-4-ene configuration, and it exhibits higher activity towards steroid substrates carrying a small or no aliphatic side chain than towards substrates having a bulky side chain at the C-17 atom. The recombinant strain could efficiently convert androst-4,9(11)-dien-3,17-dione into androst-1,4,9(11)-trien-3,17-dione (with conversion rate of 96%). 1(2)-Dehydrogenation of androst-4,9(11)-dien-3,17-dione is one of the key steps in glucocorticoid production. To the best of our knowledge, this is the first study reporting on the conversion of androst-4,9(11)-dien-3,17-dione catalyzed by recombinant KstD; the expression system of KstD_3gor_ reported here would have an impact in the industrial production of glucocorticoid in the future.

## Introduction

Significant progress has been made over the last ten years in the use of enzymes and microorganisms for the manufacturing of complex chemical compounds and replacing multi-steps chemical syntheses. Actinobacteria are known as efficient biocatalysts of steroid bioconversion since 1913 (Tak 1942). However, the recent advances in genome sequencing and bioinformatics technologies provided tools for identification of new players in cholesterol bioconversion. Although steroids are highly resistant to biodegradation, many bacteria use them as a source of carbon and energy (García et al. 2012). Microbial transformation could be carried out under mild reaction conditions with excellent yields of products and remarkable regio- and stereo-selectivity, which is hardly available for chemical synthesis. Therefore, for producing novel steroidal drugs and generating active pharmaceutical ingredients, microbial transformation is employed as a novel, efficient and economical tool (Donova 2007; García et al. 2012; Yang et al. 2015). For example, side chains of phytosterol, a byproduct from soybeans, sugar and paper industries, can be selectively degraded by a process similar to the β-oxidation of fatty acids, yielding 17-ketosteroids (Wei et al. 2010). One of the products of this degradation, 9α-hydroxy-androst-4-ene-3,17-dione, and its ∆9-analog are considered as the most important intermediates for the synthesis of corticoids such as prednisolone, betamethasone, dexamethasone, and triamcinolone (Fokina and Donova 2003; Yuan et al. 2015). The efficiency of enzymatic processes and purity of their products have obvious advantages in comparison with multi-steps chemical syntheses of hormonal drugs. However, the development of steroid biotechnology requires further studies of microorganisms able to degrade/modify steroids as well as enzymes catalyzing these reactions on the molecular level (Yang et al. 2015).

The degradation of cholesterol or its derivatives begins with the transformation of cholesterol to cholest-4-en-3-one by a cholesterol oxidase (Shao et al. 2015). The subsequent catabolism involves elimination of the alkyl side chain followed by the opening of the rings A/B and rings C/D. A 3-ketosteroid Δ1-dehydrogenase (KstD) [EC 1.3.99.4], catalyzing the elimination of the hydrogen atoms of the C-1 and C-2 in the A-ring from the polycyclic ring structure of 3-ketosteroids, is a key enzyme in microbial steroid catabolism needed for the opening of the steroid B-ring (Fernández de Las Heras et al. 2012; Zhang et al. 2013). KstD is a FAD-dependent enzyme, the natural electron acceptor appears to be vitamin K2 (Choi et al. 1995a), and they can transfer electrons to *N*-methyl phenazolium sulfate. KstDs were found in various bacteria, including *Mycobacterium* sp., *Rhodococus* sp., *Pseudomonas* sp. and *Arthrobacte*r sp. (Choi et al. 1995b; Molnar et al. 1995; van der Geize et al. 2000; Brzostek et al. 2005; Knol et al. 2008). They display a broad substrate spectrum. The KstD_1SQ1_ (KstD_1_ from *Rhodococcus erythropolis* SQ1) and KstD_2SQ1_ enzymes (KstD_2_ from *Rhodococcus erythropolis* SQ1) were specific for steroids with the 3-keto-4-ene structure such as 9α-hydroxy-androst-4-ene-3,17-dione (Knol et al. 2008). KstD_3SQ1_ (KstD_3_ from *Rhodococcus erythropolis* SQ1) had a clear preference for 3-ketosteroids with a saturated A-ring. The role of three KstDs from *Rhodococcus ruber* strain Chol-4 was studied in the steroid metabolism (Fernández de Las Heras et al. 2012).

**Fig. 1:**
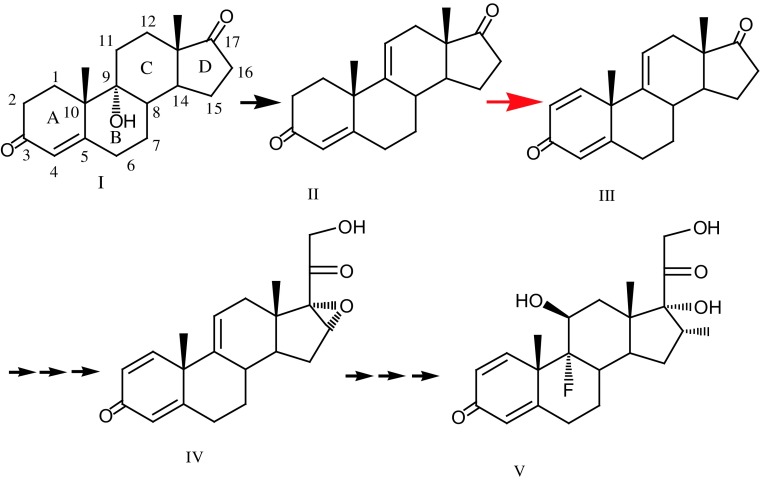
Dehydrogenation reaction in industrial production of fluorocorticoid from 9α-OH-AD. *I* 9α-hydroxy-androst-4-ene-3,17-dione; *II* androst-4,9(11)-dien-3,17-dione; *III* androst-1,4,9(11)-trien-3,17-dione; *IV* 16α,17α-epoxy-pregn-4,9(11)-dien-21-ol-3,20-dione; *V* dexamethasone

KstD proteins were expressed in *Escherichia coli* (Wei et al. 2014), *Bacillus subtilis* (Zhang et al. 2013), *Streptomyces lividans* (Choi et al. 1995a), *Rhodococcus erythropolis* (Knol et al. 2008), *Mycobacterium neoaurum* (Morii et al. 1998), etc., and used to convert androst-4-ene-3,17-dione (AD) into androsta-1,4-diene-3,17-dione (Choi et al. 1995b; Morii et al. 1998; Knol et al. 2008; Zhang et al. 2013; Wei et al. 2014). An interesting possibility to use KstD for the 1(2)-dehydrogenation of androst-4,9(11)-dien-3,17-dione [4,9(11)-AD] (a step of the pathway for glucocorticoid production, as shown in Fig. 1) has not been tested.

*Gordonia neofelifaecis* NRRL B-59395 was initially isolated from fresh faeces of a clouded leopard (*Neofelis nebulosa*) for its ability to degrade cholesterol (Liu et al. 2011). In spite of a significant number of known actinobacteria that could degrade/modify steroids, only a few genomes of them have been completely sequenced to identify all sterol catabolic genes. We sequenced *G. neofelifaecis* NRRL B-59395 genome and found 5 putative genes encoding KstD (Ge et al. 2011; Li et al. 2014). We also studied the substrate specificity of these KstDs in our former study (Zhang et al. 2015). One enzyme, KstD_3gor_, had the broadest spectrum of substrate specificity, exhibiting activity to progesterone, 16α, 17α-epoxyprogesterone and cholest-4-en-3-one.

In this work, we cloned KstD_3gor_ gene into *E. coli* vector, expressed the recombinant enzyme and characterized its enzyme specificity and selectivity. Our results indicated that KstD_3gor_ could have a possible application for the production of androst-1,4,9(11)-trien-3,17-dione in the pharmaceutical industry.

## Materials and methods

### Chemicals

Androst-4,9(11)-dien-3,17-dione with purity of 99% was obtained from Zhejiang Shengzhou pharmaceutical Co. Ltd (China). 16α,17α-epoxyprogesterone, dehydroandrosterone, were obtained from XianJu Pharmaceutical Company Ltd. (Zhejiang Province, China) with purity of 98%. Cholesterol (purity ≥99%), progesterone (purity ≥99%), 5α-cholesteran-3β-ol (with purity of 92%), 3β-hydroxypregn-5-en-20-one (with purity of 92%), phenazine methosulfate (PMS, purity ≥90%), nitro blue tetrazolium (with purity of 98%), and dimethylformamide were purchased from Sigma (USA). Cholest-4-en-3-one, androst-4-en-3,17-dione (AD), (25R)-cholesten-26-oic acid with purity of 99% were synthesized and characterized using ^1^HNMR, ^13^C NMR, IR, and TOF–MS as it was done in our previous studies (Liu et al. 2011; Wu et al. 2015). (25R)-cholesten-26-oic acid was prepared as recommended (Martin et al. 2009). Restriction enzymes, dNTPs, *Taq* polymerase were purchased from TaKaRa Co. (Dalian, China).

### Bacterial strains

*Gordonia neofelifaecis* (NRRL B-59395) was preserved in our laboratory (Ge et al. 2011) and cultivated in liquid LB medium (Luria–Bertani broth) at 37 °C. The strain had been originally isolated from the faeces of *Neofelis nebulosa*. *Escherichia coli* DH5α and *E*. *coli* BL21 (purchased from Transgen Biotech Co., Ltd, Beijing, China) were grown in LB broth or Super Optimal Broth (SOB, 2% peptone, 0.5% Yeast extract, 10 mM NaCl, 2.5 mM KCl, 10 mM MgCl_2_, 10 mM MgSO_4_) (Hanahan 1983) at 37 °C/200 rpm. Kanamycin (25 μg/mL) was added to the growth medium when necessary. For growing on solid medium, 2% (w/v) agar was added.

### Construction and expression of KstD_3gor_ in *E. coli*

The KstD_3gor_ gene was amplified from previously cloned KstD_3gor_ construct (Zhang et al. 2015) and cloned into the *Nde* I/*Bam*H I restriction sites of pET-28a(+) vector (Novagen), giving the construct pET28a-KstD3. Recombinant DNA techniques were done according to standard protocols (Sambrook and Russell 2001). *E. coli* transformation was performed as described previously (Chung et al. 1989). The DNA was isolated using the Plasmid Mini Kit (Omega, USA) according to the manufacturer’s instructions. DNA sequence was verified by sequencing service provided by the Beijing Genome Institute Sequence Facility (Shenzhen, China).

### Expression of KstD_3gor_ in *E. coli* and preparation of clarified lysate

Recombinant KstD_3gor_ protein was expressed in BL21(DE3) cells. *E. coli* cells transformed with pET28a-KstD_3_ construct were grown overnight (18 h) at 37 °C in 10 mL of LB medium containing 25 μg/mL kanamycin (the same concentration was used in all cultivation procedures) in a 50-mL Erlenmeyer flask. Ten mL of this culture was inoculated into 500 mL of LB medium containing antibiotic in a 2000-mL Erlenmeyer flask and incubated at 30 °C until the OD_600_ reached ~0.6. Then, the culture was induced with 1 mM isopropyl β-d-1-thiogalactopyranoside (IPTG), and incubated with shaking (200 rpm) at 30 °C for 6 h. The cells were harvested by centrifugation at 10,000 rpm for 10 min. The cell pellet was washed for three times with 10 mL of chilled (4 °C) 50 mM Tris–HCl buffer (pH 7.0) (every time, centrifugation was performed at 10,000 rpm for 10 min at 4 °C), resuspended in 10 mL of the same buffer and sonicated using an ultrasonic homogenizer (JY92-II, Scientz Biotechnology Co. Ltd., China) in an ice bath, with 90 cycles of 5 s on and 5 s off at 220 W. The cell lysates were centrifuged for 30 min at 18,000 rpm and 4 °C. The supernatant (clarified lysate) and the washed cell pellet were used for KstD activity gel assay or biochemical assays for the measurements of bioconversion of steroid substrates.

### KstD activity staining on native PAGE

The cell extract was used for analysis of KstD activity on native 12.5% PAGE (Knol et al. 2008). The KstD activity was visualized by incubating native gels in 100 mL 50 mM Tris–HCl buffer (pH 7.0) containing 3.1 mg phenazine methosulphate, 2.9 mg androst-4-en-3, 17-dione dissolved in ethanol and 41 mg nitroblue tetrazolium (NBT) dissolved in 70% dimethylformamide. The native gels were stained for several hours until the appearance of distinct purple-colored bands of the product of the reaction, formazan on the gel. Then, the staining was stopped by replacing the staining solution with 100 mL of 10% (v/v) acetic acid.

### Preparation of steroid-methyl-β-cyclodextrin (Me-β-CD) complex (steroid-complex)

Steroid-methyl-β-cyclodextrin (Me-β-CD) complex (steroid-complex) was prepared by the co-evaporation method as previously reported (Manosroi et al. 2008). Briefly, 0.2 mol of methyl-β-cyclodextrin and 0.1 mol of steroid substrates were dispersed in 95% ethanol and stirred with a speed of 200 rpm for 4 h at 37 °C. Then, ethanol was evaporated at 60 °C on a Buchi model R-210 rotary evaporator (0.09 Mpa, with a speed of 60 rpm at 65 °C) (The same parameters of the instrument were used throughout the study to concentrate extraction samples).

### Bioconversion of steroid substrates by KstD_3gor_-expressing *E. coli* cells and extracts

The bioconversion of steroid substrate was performed in 100 mL Erlenmeyer flasks with the clarified lysates or *E. coli* cells. To prepare *E. coli* cells, the cells expressing KstD_3gor_ were harvested by centrifugation from 4 mL fresh culture, and resuspended in 20 mL Tris–HCl buffer (pH 7.0) to the concentration of 5 × 10^7^ CFU/mL. The clarified lysates from 4 mL fresh culture were prepared as described above. Then, steroid complex was added to the 20 mL of cell suspension or clarified lysate. The final concentration of each substrate was 2 g/L. The reaction mixture was incubated at 37 °C with shaking at 200 rpm for 10–24 h. Steroids were extracted from the medium by adding the equivalent volume of ethyl acetate; after phase separation, the upper organic phase was analyzed by thin-layer chromatography (TLC). The TLC was performed on 0.25 mm-thick silica gel G (silica gel 254, Qingdao Haiyang Chemical Co., Ltd.) with cyclohexane/ethyl acetate (7:3, v/v) as the mobile phase. 10 μL sample of organic phase was applied on TLC plate. The products of the enzymatic reaction were visualized by spraying a mixture of sulfuric acid and methanol (1:6, v/v) on the plates and heating them at 100 °C until the colors developed. The extraction procedure was repeated three times, and the steroid extract was dried by reduced pressure distillation (0.09 MPa, 65 °C) and further separated by high-performance liquid chromatography (HPLC) (Shimadzu, Kyoto, Japan). To perform HPLC analysis, samples were diluted to an appropriate concentration (about 1 mg/mL) with methanol and filtered through 0.22 μm pore-size membranes. HPLC analysis was performed on an Alltima C18 column (250  ×  4.6 mm, 5 μm, Alltech, USA) under the control of an HPLC system (Shimadzu, Japan) equipped with an LC-20AB HPLC pump system and SPD-20A UV detector. The HPLC was performed using methanol–water (6:4, v/v) as the mobile phase at a flow rate of 1 mL/min.

The conversion rate of sterol was calculated according to the following formula:

$${\text{Conversion rate}} \left( \% \right) = \frac{\text{moles of product}}{\text{moles of substrate converted}}.$$

### Purification and spectroscopic analysis of transformation products

Purification of the products of bioconversion was performed as previously described (Liu et al. 2011). Briefly, 500 mL of the reaction mixture was extracted with 500 mL ethyl acetate (v/v) at room temperature for 30 min. The organic phase was collected and evaporated under reduced pressure. The crude gum was dissolved in ethyl acetate, and applied to a silica gel column (2.5 cm × 30 cm), eluted with acetone/ethyl acetate (the eluent system consisted of gradient mixtures of chloroform and acetone and ethyl acetate), at a flow rate of 1 mL/min. Fractions of 10 mL were collected. The fractions containing the same steroid intermediates were pooled and concentrated in a rotary evaporator. Finally, the product was recrystallized from anhydrous alcohol and was subjected to ^1^HNMR, ^13^C NMR, or TOF–MS analysis.

## Results

### Expression of the catalytically active KstD_3gor_ in *E. coli* cells

**Fig. 2:**
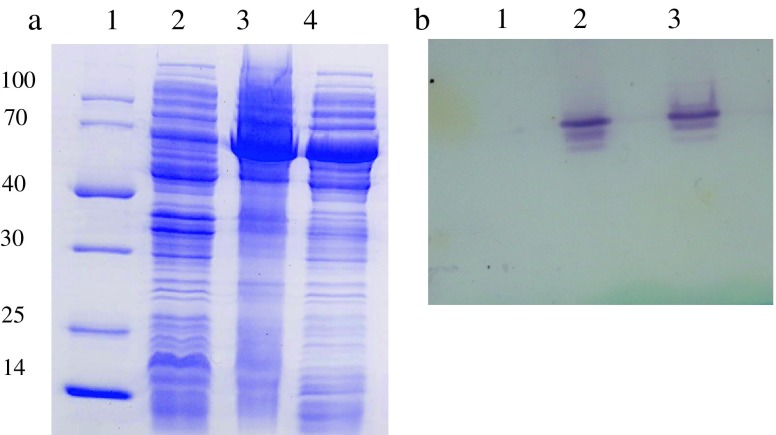
Expression of catalytically active KstD_3gor_ in BL21(DE3) cells. **a** Expression of the protein analyzed by 10% SDS-PAGE: *lane 1* molecular weight marker; *lane 2* total cell lysate of BL21(DE3)/pET28a-KstD3 before IPTG induction; *lane 3* The total cell lysate of the same culture as in *lane* 2 after IPTG induction; *lane 4*, the clarified lysate of the sample in *lane 3*. **b** KstD activity visualized on native PAGE gel loaded with total cell extracts: *lane 1* clarified lysate of BL21(DE3)/pET28a cells (negative control); *lane 2* clarified lysate of BL21(DE3) cells expressing KstD_3gor_ after 4 h induction; *lane 3* clarified lysate of the same cells as in *lane 2* after 8 h induction

To expand our knowledge about KstD_3gor_ from *G*. *neofelifaecis*, the gene of this enzyme was sub-cloned into a commercial *E. coli* vector pET28a(+) and recombinant KstD_3gor_ protein was expressed in BL21(DE3) cells. The calculated molecular mass of KstD_3gor_ (57 kDa) corresponded to the most abundant band on the 10% SDS-PAGE in the sample with induced expression of the protein (Fig. 2a, lane 3). A significant amount of KstD_3gor_ was expressed as a soluble protein (Fig. 2a, lanes 3, 4). Decreasing the temperature (from 30 to 25 °C) and increasing the induction time (from 4 to 6 h) led to a high level of recombinant protein expression.

KstD activity staining on native PAGE confirmed the expression of catalytically active enzyme. The clarified lysates were prepared and assayed by native PAGE as described in Sect. “Materials and methods”. The electron transfer from AD via phenazine methosulphate to NBT catalyzed by KstD_3gor_ resulted in the formation of a purple-colored product of the reaction, formazan (Fig. 2b, lanes 2, 3). Meanwhile, no activity was detected in the negative control sample (Fig. 2b, lane 1).

### Bioconversion of steroid substrates by KstD_3gor_-expressing *E. coli* cells and clarified lysates

**Fig. 3:**
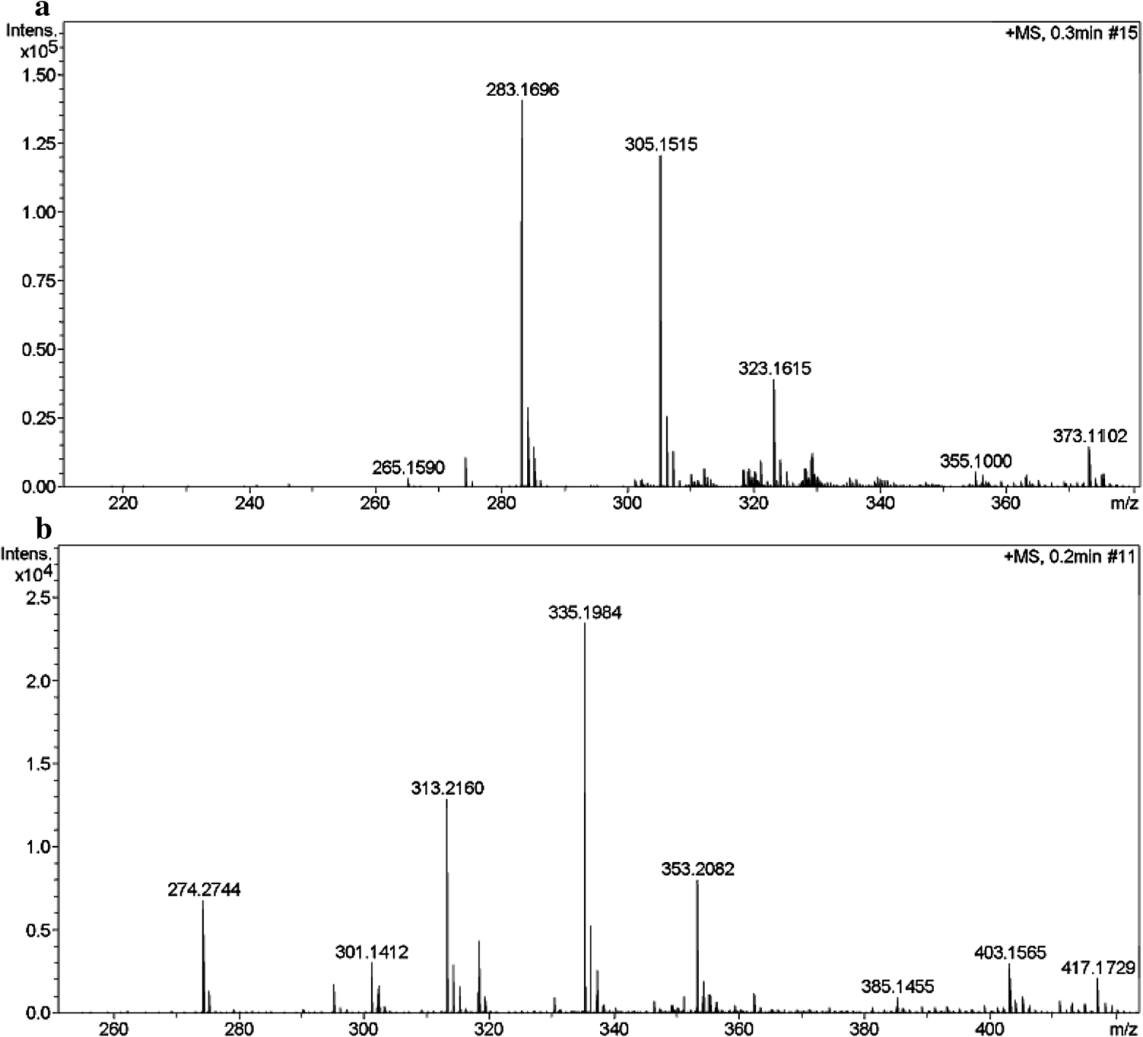
The TOF–MS spectra of the transformed product of androst-4,9(11)-dien-3,17-dione

The *E. coli* cells expressing KstD_3gor_ were tested in the dehydrogenation reaction of androst-4,9(11)-dien-3,17-dione. The structures of the products were determined using spectroscopic techniques including ^1^HNMR, ^13^C NMR and TOF–MS. The TOF–MS spectra are shown in Fig. 3a, TOF–MS *m*/*z*: 283.17[M+H]^+^, 305.15[M+Na]^+^. ^1^H NMR spectrum in CDCl_3_ (δ, ppm): 0.89 (3H, s, H-18), 1.36 (3H, s, H-19), 5.59 (br t, 1H, H-11), 6.09 (1H, s, H-4), 6.30 (1H, dd, J = 10.2, 1.9 Hz, H-2), 7.27 (1H, d, J = 10 Hz, H-1). ^13^C NMR spectrum in CDCl_3_ (δ, ppm): 154.3 (C-1), 127.2 (C-2), 186.0 (C-3), 123.8 (C-4), 166.1 (C-5), 33.7 (C-6), 31.6 (C-7), 34.2 (C-8), 143.4 (C-9), 45.9 (C-10), 119.8(C-11), 33.5 (C-12), 46.0 (C-13), 48.1 (C-14), 26.4 (C-15), 36.1 (C-16), 220.2 (C-17), 13.7 (C-18), 22.8 (C-19). The result showed that the product reduces two mass units from androst-4,9(11)-dien-3,17-dione (284.39), suggesting that dehydrogenation reaction has occurred at the C-1 and C-2 in the A-ring.

**Fig. 4:**
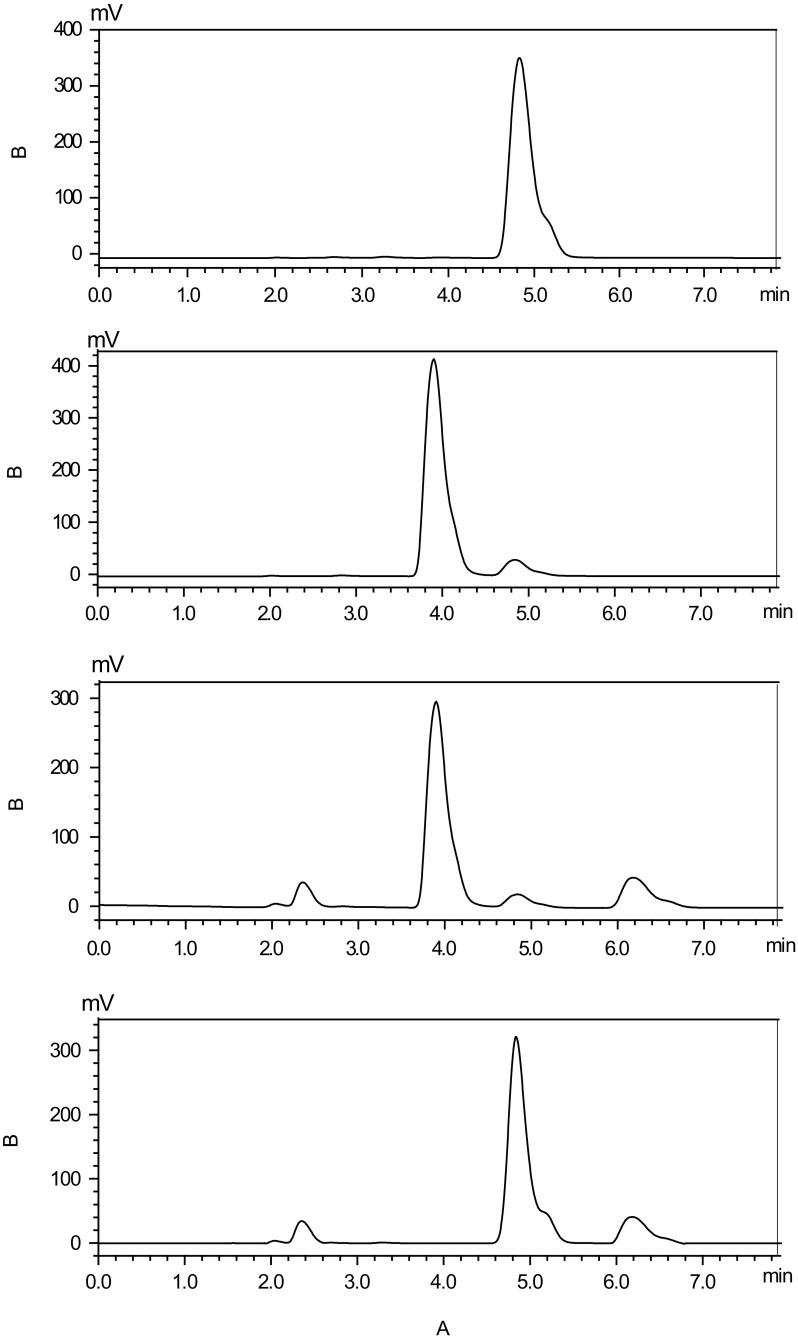
HPLC analysis of steroid substrates bioconversion by KstD_3gor_-expressing *E. coli* cells. **a** The standard samples of androst-4,9(11)-dien-3,17-dione. **b** The standard samples of androst-1,4,9(11)-trien-3,17-dione. **c** The product by KstD_3gor_-expressing *E. coli* cells. **d** The product by BL21(DE3)/pET28a cells

In a typical bioconversion experiment, 500 mL of KstD_3gor_-expressing *E. coli* cells supplemented with 2 g/L androst-4,9(11)-dien-3,17-dione produced 1.94 g/L androst-1,4,9(11)-trien-3,17-dione, the reaction mixture was incubated for 16 h, and the conversion ratio of androst-1,4,9(11)-trien-3,17-dione in the products reached the highest conversion rate (96%). The property of KstD_3gor_-expressing *E. coli* cells to catalyze the dehydrogenation of androst-1,4,9(11)-trien-3,17-dione was confirmed by HPLC using androst-4,9(11)-dien-3,17-dione as a substrate (as shown in Fig. 4). The similar results were obtained for the clarified lysates (data not shown).

### Substrate preference and selectivity of KstD3_gor_

To characterize the substrate preferences of the recombinant KstD_3gor_, nine steroid substrates were used in the dehydrogenation reaction catalyzed by KstD_3gor_-expressing cells (Table 1). The structures of the products were determined using spectroscopic techniques, The TOF–MS spectra of the transformed products from progesterone are shown in Fig. 3b, TOF–MS *m*/*z*: 313.21 [M+H]^+^, 335.19[M+Na]^+^. ^1^H NMR spectrum in CDCl3 (δ, ppm): 0.70 (3H, s, H-18), 1.24 (3H, s, H-19), 2.13 (3H, s, H-21), 6.09 (1H, s, H-4), 6.30 (1H, dd, J = 10.2, 1.9 Hz, H-2), 7.04 (1H, d, J = 10 Hz, H-1). ^13^C NMR spectrum in CDCl_3_ (δ, ppm): 155.6 (C-1), 127.4 (C-2), 186.3 (C-3), 123.6 (C-4), 168.9 (C-5), 33.5 (C-6), 31.1 (C-7), 35.3 (C-8), 55.4 (C-9), 38.5 (C-10), 22.7 (C-11), 42.9 (C-12), 43.9 (C-13), 51.9 (C-14), 22.8 (C-15), 32.5 (C-16), 62.9 (C-17), 13.8 (C-18), 18.7 (C-19), 209.2 (C-20), 24.5 (C-21). The ^1^HNMR, ^13^C NMR, and TOF–MS spectroscopic data suggested that progesterone (pregn-4-ene-3,20-dione) was transformed to pregn-1,4-diene-3,20-dione. The transformed products of androst-4-ene-3,17-dione and 16α,17α-epoxyprogesterone were identified as androst-1,4-diene-3,17-dione and 16α,17-epoxypregn-1,4-diene-3,20-dione, respectively; the spectroscopic data were as the same as our previous reported work (Liu et al. 2011; Zhang et al. 2015).

**Table 1:** Bioconversions of different kinds of steroids by recombinant strain KstD_3_

Steroid substrate	Transformation rate (%)
Androst-4-ene-3,17-dione	96.21 ± 2
4,9(11)-Androstadiene-3,17-dione	96.35 ± 2
Cholest-4-en-3-one	0
Progesterone	90.12 ± 2
16α,17α-epoxyprogesterone	25.11 ± 2
(25R)-Cholesten-26-oic acids	0
Dehydroandrosterone	0
5α-Cholesteran-3β-ol	0
5-Pregnen-3β-ol-20-one	0

**Fig. 5:**
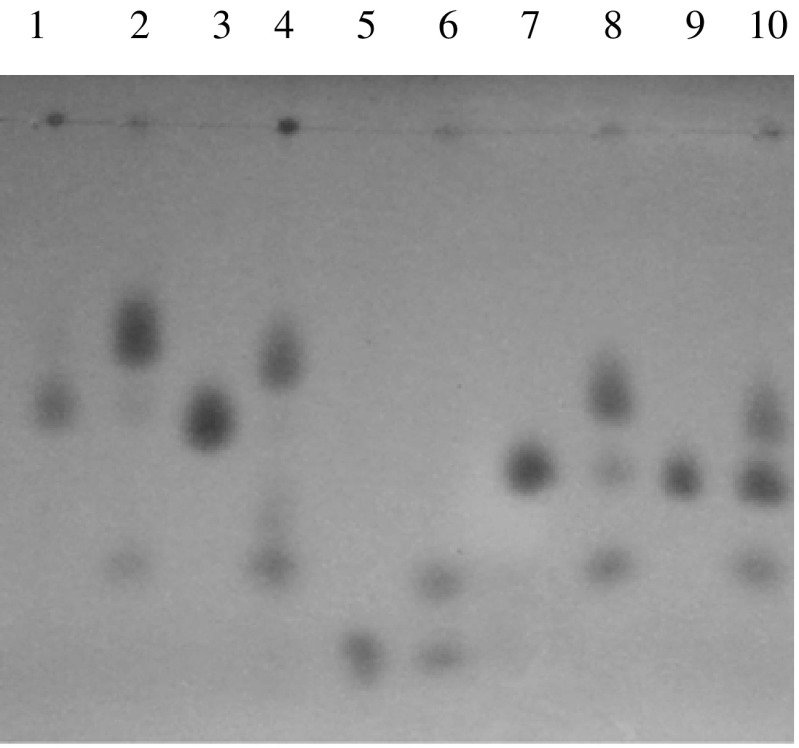
TLC analysis of several steroid substrates conversion by KstD_3gor_-expressing cells. *Lane 1* androst-4-en-3, 17-dione; *lane 2* the conversion products of androst-4-en-3, 17-dione; *lane 3* androst-4,9(11)-dien-3,17-dione; *lane 4* the conversion products of androst-4,9(11)-dien-3,17-dione; *lane 5* cholest-4-en-3-one; *lane 6* the conversion products of cholest-4-en-3-one; *lane 7* progesterone; *lane 8* the conversion products of progesterone; *lane 9* 16α,17α-epoxyprogesterone; *lane 10* the conversion products of progesterone 16α,17α-epoxyprogesterone

As shown in Table 1, KstD_3gor_-expressing cells exhibited the dehydrogenase activity towards androst-4-en-3,17-dione, androst-4,9(11)-dien-3,17-dione, progesterone and 16α,17α-epoxyprogesterone (Fig. 5). Androst-4-en-3,17-dione, androst-4,9(11)-dien-3,17-dione appear to be the preferred KstD_3gor_ substrate, with conversion rate of 96%, respectively. However, KstD_3gor_-expressing cells had no ability of catalyzing the conversion of cholest-4-en-3-one, (25R)-cholesten-26-oic acids, dehydroandrosterone, 5α-cholesteran-3β-ol, 3β-hydroxypregn-5-en-20-one. No KstD activity was observed in negative control (data not shown).

## Discussion

KstDs are widely found in actinobacteria as they play an important role in microbial steroid degradation. Different isoforms of the enzyme have been found exhibiting different substrate specificity and having different roles that could be strain-dependent (Knol et al. 2008). The genome of *R. ruber* strain Chol-4 contains three different KstD genes, and two of them contribute to cholesterol catabolism (Fernández de Las Heras et al. 2012). *Rhodococcus jostii* RHA1 KstDs display a quite different substrate specificity (Knol et al. 2008).

The sterol catabolic genes are highly conserved in *G. neofelifaecis*, *R. jostii* RHA1, and *Mycobacterium tuberculosis*, and mainly organized in three specific clusters. In our previous research, the substrate preference of five *G. neofelifaecis* KstDs was investigated preliminarily (Zhang et al. 2015). Here, the KstD_3gor_ was biochemically characterized in detail.

**Fig. 6:**
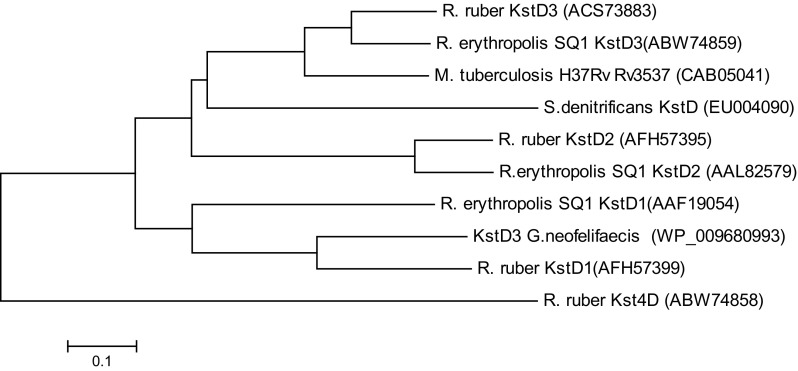
Phylogenetic tree of characterized bacterial KstDs. MEGA version 5.0 was used for the construction of a phylogenetic tree using the amino acid sequences of characterized bacterial KstDs

A phylogenetic tree (Fig. 6) of the characterized KstDs revealed that they share high similarity with other bacterial KstD homolog. They clustered into at least four distinct groups (Knol et al. 2008). KstD_3gor_ showed 63.6 and 44.7% amino acid identity with *R. ruber* KstD_1_ and *R. erythropolis* KstD_1_, correspondingly. Both KstD_3gor_ and *R. erythropolis* KstD_1_ prefer 3-ketosteroids with a saturated A-ring as substrates.

Since KstD_3gor_ activity was not detected with trans-dehydroandrosterone which has no aliphatic side chain, the 3-keto-4-ene configuration of the steroid substrate appears to be important for KstD_3gor_ activity. KstD_3gor_ efficiently catalyzed the Δ^1^-dehydrogenation of steroid substrates carrying a small or no aliphatic side chain, compared to the steroids with a bulky side chain at the C-17 atom (i.e., cholest-4-en-3-one, (25R)-cholesten-26-oic acid). The conversion rate of 16α,17α-epoxyprogesterone was about 3.5-fold lower compared to that of progesterone, showing that besides the length of the 17-alkyl side chain, the additional molecular group in D-ring affects the catalytic activity of KstD_3gor_. The 4,9(11)-dehydrogenation of C-ring had no effect on the KstD_3gor_ activity.

Cholest-4-en-3-one is the second intermediate product of cholesterol catabolism (Knol et al. 2008), and (25R)-cholesten-26-oic acids are the first product of the side-chain degradation, but KstD_3gor_, KstD_1-3SQ1_ and KstD_H37Rv_ could not catalyze their 1(2)-dehydrogenation. Thus, these KstDs could act after elimination of the sterol side-chain.

The introduction of the C1-C2 double bond into ring A of steroid could improve the biological activity of the original steroid substrate (i.e., prednisone and prednisolone) (Fokina and Donova 2003). The enzyme KstD catalyzing this reaction has been widely characterized during the past two decades. We studied the KstD_3gor_ 1(2)-dehydrogenation activity for steroids with different A-ring, B-ring, C-ring and D-ring modifications. The results showed that KstD3gor has high activity towards both 4-AD and 4-pregnene-3,20-dione (progesterone) which contain 3-keto-4-ene configuration and a small or no aliphatic side chain at the C-17 atom. It appears to be different from the KstD_1SQ1_ (KstD_1_ from *Rhodococcus erythropolis* SQ1) which shows high activity towards 4-AD but low activity towards 4-pregnene-3,20-dione (Knol et al. 2008). While KstD_3SQ1_ had a clear preference for 3-ketosteroids with a saturated A-ring, the 1(2)-dehydrogenation of androst-4,9(11)-dien-3,17-dione is one step of the pathway for glucocorticoid production (Fig. 1). This pathway is considered to be an effective method to produce fluorocorticoids (such as dexamethasone, betamethasone, triamcinolone, etc.) and other valuable steroid drugs (Fokina and Donova 2003). But the application of KstD for the 1(2)-dehydrogenation of androst-4,9(11)-dien-3,17-dione [4,9(11)-AD] has not been tested. The conversion rate of androst-4,9(11)-dien-3,17-dione to androst-1,4,9(11)-trien-3,17-dione by the KstD_3gor_-expressing cells was more than 96%. Thus, this work provided a candidate, KstD_3gor_-expressing cells, for the industrial conversion of steroids to their 1(2)-dehydro-analogs.

In summary, the KstD_3gor_ from *G. neofelifaecis* was expressed efficiently in a soluble form in *E. coli*. The KstD_3gor_-expressing cells effectively converted androst-4,9(11)-dien-3,17-dione and androst-4-en-3, 17-dione to androst-1,4,9(11)-trien-3,17-dione and androst-1,4-en-3,17-dione, respectively. Both of them can be recommended as substrate for 1(2)-dehydrogenation by KstD_3gor_-expressing *E. coli* in the commercial production of 9-halogenated steroids.
